# Effects of acupuncture for nonalcoholic fatty liver disease: A protocol for systematic review and meta-analysis

**DOI:** 10.1097/MD.0000000000023219

**Published:** 2020-11-20

**Authors:** Xiaoming Zang, Mi Sun, Jin Xian, Huijuan Yu, Xin Zhang, Changyun Zhang, Qiwen Tan

**Affiliations:** a Shandong University of Traditional Chinese Medicine; b Shandong Provincial Hospital of Traditional Chinese Medicine, Shandong, China.

**Keywords:** Nonalcoholic fatty liver disease (NAFLD), acupuncture, protocol, systematic review and meta-analysis

## Abstract

**Background:**

Nonalcoholic fatty liver disease (NAFLD) is a multisystem disease, which may develop gradually into nonalcoholic steatohepatitis, liver fibrosis, and cirrhosis. As a classical method of traditional Chinese medicine, acupuncture has been used to treat NAFLD in clinical practice. However, there is no systematic review and meta-analysis of acupuncture on NAFLD. There is an urgent need to evaluate the clinical efficacy of acupuncture for NAFLD.

**Methods:**

We will perform a comprehensive retrieval in the following electronic databases: MEDLINE, Embase, Cochrane Library Central Register of Controlled Trials, PubMed, Science Citation Index Expanded (Web of Science), Epistemonikos, China National Knowledge Infrastructure (CNKI) database, Wanfang Data Knowledge Service Platform, Chinese Scientific Journals Database (VIP), Chinese Biomedical Literature Service System (SinoMed), and other databases from their inception to October 2020. We will select the qualified studies for data extraction and assess the quality and risk of bias, independently. The meta-analyses will be conducted by using the RevMan 5.3.

**Results:**

This study will provide a reliable basis for the treatment of NAFLD with acupuncture.

**Conclusion:**

The findings will be an available reference to evaluate whether acupuncture is an effective intervention for patient with NAFLD.

**OSF registration number:**

10.17605/OSF.IO/VFYXH.

## 1 Introduction

Nonalcoholic fatty liver disease (NAFLD) is a multisystem disease associated with obesity, insulin resistance, and dyslipidemia, which may develop gradually into nonalcoholic steatohepatitis, liver fibrosis, and cirrhosis. It has become one of the main causes of cirrhosis and hepatocellular carcinoma in recent years.^[1]^ Moreover, NAFLD is a risk factor for many diseases, including cardiovascular disease,^[2]^ type 2 diabetes mellitus,^[3]^ chronic kidney disease,^[4]^ and cancer.^[5]^ In addition, NAFLD is associated with a high incidence of chronic diseases such as obstructive sleep apnea, polycystic ovarian syndrome, colorectal polyps, osteoporosis, and stroke.^[6]^ Thus, early and appropriate prevention and treatment interventions are needed in the management of NAFLD.

Acupuncture is an ancient Chinese medicine-based approach, which has been widely used for the prevention and treatment of various diseases. As a classical method of traditional Chinese medicine, acupuncture has been applied to treat NAFLD in clinical practice due to its advantages of low cost, few side effects, and simple operation. A clinical study has demonstrated that acupuncture can effectively treat NAFLD and present better therapeutic effect on hepatic fat status, glycolipid metabolism, and insulin resistance.^[7]^ Several experiments conducted in NAFLD models showed that acupuncture could repress the process of NAFLD by inhibiting inflammation, reducing oxidative stress, and promoting lipid metabolism in liver cells.^[8,9]^ Moreover, acupuncture at Zu san li, Guan yuan, and Yong quan could suppress lipid absorption by downregulating the expression of apolipoproteins in the small intestine.^[10]^ Although the proportion of application of acupuncture for NAFLD is increasing, there is no systematic review and meta-analysis of acupuncture on NAFLD. Hereby, we will systematically evaluate the clinical efficacy of acupuncture for NAFLD, so as to provide an objective and scientific basis for clinical practice.

## 2 Objective

This systematic review aims to identify and critically summarize randomized controlled trials (RCTs) of acupuncture for treating NAFLD. A comprehensive understanding of the current level of evidence in this work will provide evidence to judge whether acupuncture is an effective intervention for patient with NAFLD.

## 3 Methods and analysis

### 3.1 Study design

This work will be conducted followed the guideline of the Preferred Reporting Items for Systematic Review and Meta-Analysis Protocols (PRISMA-P) recommendations.^[11]^ We have registered this work at Open Science Framework (OSF, https://osf.io/), an open source project management that helps in the design of studies. The registration DOI of this study is 10.17605/OSF.IO/VFYXH.

### 3.2 Types of studies

We will include RCTs that used acupuncture or a combination of acupuncture and routine pharmacotherapy as treatment measures. Nonrandomized control studies and observational study will be excluded in the review. Language will be limited to English and Chinese.

### 3.3 Type of participants

We will include patients with a diagnosis of NAFLD based on liver histology or imaging (ultrasound, computer tomography, magnetic resonance imaging). There will be no limitation about age, gender, region, and other factors.

### 3.4 Types of interventions

- Experimental intervention: we will only include studies that interventions involved acupuncture alone or combined with other routine pharmacotherapy, as well as those with control groups, which can verify the therapeutic effect of acupuncture.
- Control intervention: trials in which the control group will include no treatment, placebo, exercise intervention, diet intervention, and conventional treatments. Conventional treatments include drugs recommended by the international or domestic authorized clinical guidelines.

### 3.5 Types of outcome measures

#### 3.5.1 Primary outcomes

The improvement of imaging markers, liver enzymes, serological indexes of hepatic fibrosis, and serum NAFLD liver fat score.

#### 3.5.2 Secondary outcomes

Secondary outcomes include the changes of BMI, insulin levels, lipid profiles, total efficacy rate, and adverse events.

### 3.6 Search methods for identification of studies

#### 3.6.1 Electronics searches

We will identify trials through systematic searches of the following bibliographic databases: MEDLINE, Embase, Cochrane Library Central Register of Controlled Trials, PubMed, Science Citation Index Expanded (Web of Science), Epistemonikos, China National Knowledge Infrastructure (CNKI) database, Wanfang Data Knowledge Service Platform, Chinese Scientific Journals Database (VIP), Chinese Biomedical Literature Service System (SinoMed) from their inception to October 2020. The reference lists of relevant records will also be reviewed to identify potentially eligible trials.

#### 3.6.2 Searching other resources

- Google scholar and Baidu scholar.
- WHO International Clinical Trial Registry Platform.
- Chinese Clinical Trial Registry (ChiCTR).
- ClinicalTrials.gov.

### 3.7 Data collection and analysis

#### 3.7.1 Selection of studies

**Figure 1:**
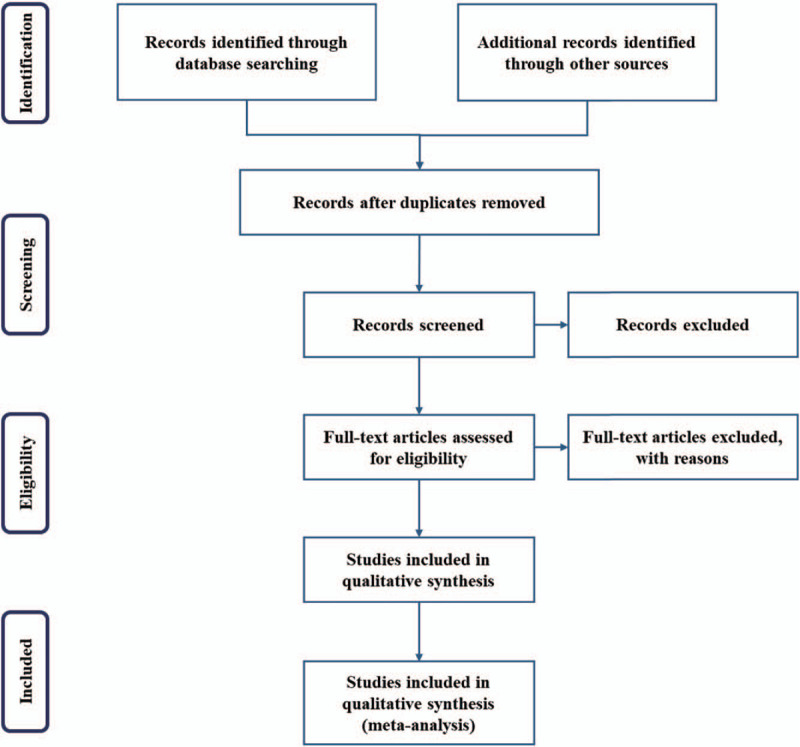
Flow chart of study selection.

The titles and abstracts of all searched studies will be assessed independently by 2 methodological trained reviewers in accordance with the established selection criteria. We will assess all potentially relevant articles as full-text. Any disagreements generated between the 2 reviewers will be solved through consensus with other authors. A PRISMA flow diagram will be drawn to illustrate the study selection procedure (Fig. 1).

#### 3.7.2 Data extraction and management

Two review authors will independently extract information from all eligible studies. The extracted information will be export to Excel table, which includes the first authors of the article, publication year, interventions in experimental group and control group, time of treatment, sample size, age, gender, outcome indicators, and adverse events. If data are not available from these studies, we will contact the corresponding authors to obtain the information.

#### 3.7.3 Measures of treatment effect

We will use RevMan V5.3 (the Nordic Cochrane Centre, Copenhagen, Denmark) to analyze the data. For dichotomous variables, a risk ratio (RR) with 95% confidence interval (95% CI) will be used for analysis. For continuous outcomes, we will calculate the mean differences (MDs) and the standard mean difference (SMD) with 95% CI to evaluate the treatment effect.

#### 3.7.4 Assessment of risk of bias

Two authors will independently assess risk of bias for each study using the Cochrane Collaboration's tool.^[12]^ We will evaluate the methodology through 6 items, including performance bias, detection bias, attrition bias, reporting bias, and other sources of bias. The assessment will be classified into 3 levels: “Low risk,” “High risk,” or “Unclear risk.” Any disagreements between the 2 reviewers will be resolved by discussion and consensus among all authors.

#### 3.7.5 Dealing with missing data

When data are not available from the studies, the missing information will be obtained by contacting the corresponding authors. Then, we will perform a sensitivity analysis using imputations of missing outcome data of dichotomous outcomes in best-worse and worse-best case scenarios to assess the potential impact of loss to follow-up.^[13,14]^

#### 3.7.6 Assessment of heterogeneity

The Cochrane *X*^*2*^ and *I*^*2*^ tests will be utilized to assess the statistical heterogeneity of evidence.^[15]^ When *P* ≥ .1 and *I*^2^ ≤ 50%, it is considered that there is no statistical heterogeneity or the heterogeneity is small. When *P* < .1 and *I*^2^ > 50%, the result indicates that there is a statistical heterogeneity.

#### 3.7.7 Data analysis

Review Manager software version 5.3 will be used for data synthesis and analysis. If there is no heterogeneity, the data are synthesized using a fixed effect model. If there is significant heterogeneity, a random effect model is used to analyze.

#### 3.7.8 Sensitivity analysis

We will conduct a sensitivity analysis will be performed to evaluate the robustness of the results. We will remove the low-level quality study one by one and then compile the data to assess the impact of sample size, study quality, statistical method, and missing data on the result of this work.

#### 3.7.9 Assessment of reporting bias

A funnel plot will be drawn to assess the publication bias if we include more than 10 studies. The potential reporting biases will be statistical appraised by the Egger test.^[16]^

#### 3.7.10 Grading the quality of evidence

The Grading of Recommendations Assessment, Development and Evaluation (GRADE) will be used to assess the quality level of evidence.^[17]^ The assessments of evidence quality will be assorted into “high,” “moderate,” “low,” and “very low” quality.^[18]^

### 3.8 Patient and public involvement

Patient and public were not involved in this study.

### 3.9 Ethics and dissemination

No ethical approval will be required because the data used are not linked to individual patient. The results of this review will be published in a peer-reviewed journal.

## 4 Discussion

As one of the most common cause of chronic liver disease in many countries worldwide, NAFLD is a condition in which there is accumulation of excess fat in the liver of people who drink little or no alcohol. At present, there is no effective treatment available in the therapy of NAFLD.^[19]^ Thus, it is of significance to seek new therapies for the treatment of NAFLD. Acupuncture has been used to treat NAFLD through regulating lipid metabolism, participating lipid metabolism-related signaling pathways, improving insulin resistance, and increasing the antioxidant levels of liver tissue.^[20,21]^ In this work, we provide a detailed summary of the current evidence related to the efficacy of acupuncture in treating the patients with NAFLD. The results of this review will be useful to clinicians regarding the use of acupuncture in NAFLD treatment.

## Author contributions

**Conceptualization:** Qiwen Tan.

**Data curation:** Xiaoming Zang, Mi Sun.

**Formal analysis:** Xiaoming Zang, Mi Sun.

**Funding acquisition:** Qiwen Tan.

**Methodology:** Huijuan Yu, Changyun Zhang.

**Resources:** Xiaoming Zang, Mi Sun, Jin Xian, Huijuan Yu

**Software:** Xiaoming Zang, Mi Sun

**Supervision:** Qiwen Tan, Jin Xian, Xin Zhang, Changyun Zhang.

**Validation:** Xiaoming Zang, Huijuan Yu.

**Visualization:** Xiaoming Zang, Jin Xian, Xin Zhang.

**Writing - original draft:** Xiaoming Zang, Mi Sun

**Writing – review & editing:** Xin Zhang, Changyun Zhang, Qiwen Tan.

## References

[1] Kanwal F Kramer JR Mapakshi S Risk of hepatocellular cancer in patients with non-alcoholic fatty liver disease. Gastroenterology 2018;155:1828–37.e2. 30144434 10.1053/j.gastro.2018.08.024 PMC6279617

[2] Targher G Day CP Bonora E Risk of cardiovascular disease in patients with nonalcoholic fatty liver disease. N Engl J Med 2010;363:1341–50. 20879883 10.1056/NEJMra0912063

[3] Tanase DM Gosav EM Costea CF The intricate relationship between type 2 diabetes mellitus (T2DM), insulin resistance (IR), and nonalcoholic fatty liver disease (NAFLD). J Diabetes Res 2020;2020:3920196. 32832560 10.1155/2020/3920196 PMC7424491

[4] Park H Dawwas GK Liu X Nonalcoholic fatty liver disease increases risk of incident advanced chronic kidney disease: a propensity-matched cohort study. J Intern Med 2019;286:711–22. 31359543 10.1111/joim.12964 PMC6851415

[5] Park H Dawwas GK Liu X Nguyen MH Nonalcoholic fatty liver disease increases risk of incident advanced chronic kidney disease: a propensity-matched cohort study. J Intern Med 2019;286:711–22. 31359543 10.1111/joim.12964 PMC6851415

[6] Kumar R Priyadarshi RN Anand U Non-alcoholic fatty liver disease: growing burden, adverse outcomes and associations. J Clin Transl Hepatol 2020;8:76–86. 32274348 10.14218/JCTH.2019.00051 PMC7132013

[7] Dong C Zhang C-R Xue B-Y Electroacupuncture combined with lifestyle control on obese nonalcoholic fatty liver disease: a randomized controlled trial. Zhongguo Zhen Jiu 2020;40:129–34. 32100496 10.13703/j.0255-2930.20190201-k00034

[8] Zhu L-L Wei W-M Zeng Z-H Impact of electro-acupuncture on lipid metabolism in rats with non-alcoholic fatty liver disease. Sichuan Da Xue Xue Bao Yi Xue Ban 2012;43:847–50. 23387211

[9] Meng X Guo X Zhang J Acupuncture on ST36, CV4 and KI1 suppresses the progression of methionine- and choline-deficient diet-induced nonalcoholic fatty liver disease in mice. Metabolites 2019;9:299. 10.3390/metabo9120299 PMC6949943 31835339

[10] Han J Guo X Meng X-J Acupuncture improved lipid metabolism by regulating intestinal absorption in mice. World J Gastroenterol 2020;26:5118–29. 32982113 10.3748/wjg.v26.i34.5118 PMC7495030

[11] Moher D Shamseer L Clarke M Preferred reporting items for systematic review and meta-analysis protocols (PRISMA-P) 2015 statement. Syst Rev 2015;4:1. 25554246 10.1186/2046-4053-4-1 PMC4320440

[12] Higgins JPT Altman DG Gøtzsche PC The Cochrane Collaboration's tool for assessing risk of bias in randomised trials. BMJ 2011;343:d5928. 22008217 10.1136/bmj.d5928 PMC3196245

[13] Akl EA Kahale LA Agoritsas T Handling trial participants with missing outcome data when conducting a meta-analysis: a systematic survey of proposed approaches. Syst Rev 2015;4:98. 26202162 10.1186/s13643-015-0083-6 PMC4511978

[14] Marker S Barbateskovic M Perner A Prophylactic use of acid suppressants in adult acutely ill hospitalised patients: a systematic review with meta-analysis and trial sequential analysis. Acta Anaesthesiol Scand 2020;64:714–28. 32060905 10.1111/aas.13568

[15] Higgins JPT Thompson SG Quantifying heterogeneity in a meta-analysis. Stat Med 2002;21:1539–58. 12111919 10.1002/sim.1186

[16] Peters JL Sutton AJ Jones DR Contour-enhanced meta-analysis funnel plots help distinguish publication bias from other causes of asymmetry. J Clin Epidemiol 2008;61:991–6. 18538991 10.1016/j.jclinepi.2007.11.010

[17] Group GW Grading quality of evidence and strength of recommendations. BMJ 2004;328:1490. 15205295 10.1136/bmj.328.7454.1490 PMC428525

[18] Guyatt GH Oxman AD Schünemann HJ GRADE guidelines: a new series of articles in the Journal of Clinical Epidemiology. J Clin Epidemiol 2011;64:380–2. 21185693 10.1016/j.jclinepi.2010.09.011

[19] Taheri H Malek M Ismail-Beigi F Effect of empagliflozin on liver steatosis and fibrosis in patients with non-alcoholic fatty liver disease without diabetes: a randomized, double-blind, placebo-controlled trial. Adv Ther 2020;37:4697–708. 32975679 10.1007/s12325-020-01498-5 PMC7547956

[20] Gao Y Chen R Liang F Mechanisms of acupuncture for non-alcoholic fatty liver disease: researches progress and prospects. Zhongguo Zhen Jiu 2018;38:109–13. 29354946 10.13703/j.0255-2930.2018.01.028

[21] Liang F Koya D Acupuncture: is it effective for treatment of insulin resistance? Diabetes Obes Metab 2010;12:555–69. 20590731 10.1111/j.1463-1326.2009.01192.x

